# Supplementary material for: Pyruvate Dehydrogenase Kinase 1 inhibition mediated oxidative phosphorylation enhancement in cartilage promotes osteoarthritis progression
Source: BMC Musculoskelet Disord. 2023 Jul 20;24:597. doi: 10.1186/s12891-023-06585-6 (PMC10357736; doi:10.1186/s12891-023-06585-6)
Supplement: Supplementary file 1 — Additional file 1. [file 12891_2023_6585_MOESM1_ESM.zip › KEGG.Gsea.1653622432464/KEGG_AMYOTROPHIC_LATERAL_SCLEROSIS_ALS.html]

Details for gene set KEGG\_AMYOTROPHIC\_LATERAL\_SCLEROSIS\_ALS[GSEA]

|  || Dataset | 55457\_collapsed\_to\_symbols.cls.cls #OA\_versus\_normal.cls.cls #OA\_versus\_normal\_repos |
| Phenotype | cls.cls#OA\_versus\_normal\_repos |
| Upregulated in class | normal |
| GeneSet | KEGG\_AMYOTROPHIC\_LATERAL\_SCLEROSIS\_ALS |
| Enrichment Score (ES) | -0.3228774 |
| Normalized Enrichment Score (NES) | -1.2106012 |
| Nominal p-value | 0.19422151 |
| FDR q-value | 0.34520593 |
| FWER p-Value | 1.0 |
Table: GSEA Results Summary

  

Fig 1: Enrichment plot: KEGG\_AMYOTROPHIC\_LATERAL\_SCLEROSIS\_ALS      
 Profile of the Running ES Score & Positions of GeneSet Members on the Rank Ordered List

  

| SYMBOL | TITLE | RANK IN GENE LIST | RANK METRIC SCORE | RUNNING ES | CORE ENRICHMENT || 1 | CASP1 | caspase 1 [Source:HGNC Symbol;Acc:HGNC:1499] | 431 | 0.694 | 0.0124 | No |
| 2 | GRIN2A | glutamate ionotropic receptor NMDA type subunit 2A [Source:HGNC Symbol;Acc:HGNC:4585] | 1054 | 0.506 | -0.0024 | No |
| 3 | NEFL | neurofilament light chain [Source:HGNC Symbol;Acc:HGNC:7739] | 1239 | 0.469 | 0.0143 | No |
| 4 | CAT | catalase [Source:HGNC Symbol;Acc:HGNC:1516] | 1731 | 0.394 | 0.0022 | No |
| 5 | TNF | tumor necrosis factor [Source:HGNC Symbol;Acc:HGNC:11892] | 2098 | 0.350 | -0.0031 | No |
| 6 | GRIA2 | glutamate ionotropic receptor AMPA type subunit 2 [Source:HGNC Symbol;Acc:HGNC:4572] | 2342 | 0.322 | -0.0006 | No |
| 7 | MAPK14 | mitogen-activated protein kinase 14 [Source:HGNC Symbol;Acc:HGNC:6876] | 2352 | 0.320 | 0.0199 | No |
| 8 | SOD1 | superoxide dismutase 1 [Source:HGNC Symbol;Acc:HGNC:11179] | 2413 | 0.313 | 0.0359 | No |
| 9 | MAPK13 | mitogen-activated protein kinase 13 [Source:HGNC Symbol;Acc:HGNC:6875] | 2544 | 0.298 | 0.0456 | No |
| 10 | CCS | copper chaperone for superoxide dismutase [Source:HGNC Symbol;Acc:HGNC:1613] | 2727 | 0.278 | 0.0498 | No |
| 11 | CASP3 | caspase 3 [Source:HGNC Symbol;Acc:HGNC:1504] | 3202 | 0.230 | 0.0282 | No |
| 12 | PRPH2 | peripherin 2 [Source:HGNC Symbol;Acc:HGNC:9942] | 3770 | 0.179 | -0.0040 | No |
| 13 | BAD | BCL2 associated agonist of cell death [Source:HGNC Symbol;Acc:HGNC:936] | 3788 | 0.178 | 0.0064 | No |
| 14 | PPP3CA | protein phosphatase 3 catalytic subunit alpha [Source:HGNC Symbol;Acc:HGNC:9314] | 3803 | 0.176 | 0.0170 | No |
| 15 | DERL1 | derlin 1 [Source:HGNC Symbol;Acc:HGNC:28454] | 3855 | 0.170 | 0.0243 | No |
| 16 | PRPH | peripherin [Source:HGNC Symbol;Acc:HGNC:9461] | 3982 | 0.157 | 0.0249 | No |
| 17 | NOS1 | nitric oxide synthase 1 [Source:HGNC Symbol;Acc:HGNC:7872] | 4228 | 0.134 | 0.0148 | No |
| 18 | PPP3CB | protein phosphatase 3 catalytic subunit beta [Source:HGNC Symbol;Acc:HGNC:9315] | 5311 | 0.046 | -0.0663 | No |
| 19 | TP53 | tumor protein p53 [Source:HGNC Symbol;Acc:HGNC:11998] | 5511 | 0.032 | -0.0797 | No |
| 20 | BCL2 | BCL2 apoptosis regulator [Source:HGNC Symbol;Acc:HGNC:990] | 5609 | 0.023 | -0.0857 | No |
| 21 | BID | BH3 interacting domain death agonist [Source:HGNC Symbol;Acc:HGNC:1050] | 5715 | 0.014 | -0.0930 | No |
| 22 | BCL2L1 | BCL2 like 1 [Source:HGNC Symbol;Acc:HGNC:992] | 6698 | -0.055 | -0.1657 | No |
| 23 | SLC1A2 | solute carrier family 1 member 2 [Source:HGNC Symbol;Acc:HGNC:10940] | 7026 | -0.082 | -0.1857 | No |
| 24 | PPP3CC | protein phosphatase 3 catalytic subunit gamma [Source:HGNC Symbol;Acc:HGNC:9316] | 7031 | -0.082 | -0.1806 | No |
| 25 | RAC1 | Rac family small GTPase 1 [Source:HGNC Symbol;Acc:HGNC:9801] | 7192 | -0.096 | -0.1867 | No |
| 26 | MAP2K6 | mitogen-activated protein kinase kinase 6 [Source:HGNC Symbol;Acc:HGNC:6846] | 7246 | -0.099 | -0.1843 | No |
| 27 | RAB5A | "RAB5A, member RAS oncogene family [Source:HGNC Symbol;Acc:HGNC:9783]" | 8049 | -0.159 | -0.2361 | No |
| 28 | CASP9 | caspase 9 [Source:HGNC Symbol;Acc:HGNC:1511] | 8142 | -0.166 | -0.2323 | No |
| 29 | MAPK12 | mitogen-activated protein kinase 12 [Source:HGNC Symbol;Acc:HGNC:6874] | 8161 | -0.168 | -0.2226 | No |
| 30 | TNFRSF1A | TNF receptor superfamily member 1A [Source:HGNC Symbol;Acc:HGNC:11916] | 8196 | -0.171 | -0.2139 | No |
| 31 | CYCS | "cytochrome c, somatic [Source:HGNC Symbol;Acc:HGNC:19986]" | 8410 | -0.186 | -0.2181 | No |
| 32 | NEFH | neurofilament heavy chain [Source:HGNC Symbol;Acc:HGNC:7737] | 8458 | -0.190 | -0.2092 | No |
| 33 | CHP2 | calcineurin like EF-hand protein 2 [Source:HGNC Symbol;Acc:HGNC:24927] | 8573 | -0.199 | -0.2048 | No |
| 34 | NEFM | neurofilament medium chain [Source:HGNC Symbol;Acc:HGNC:7734] | 8763 | -0.213 | -0.2054 | No |
| 35 | DAXX | death domain associated protein [Source:HGNC Symbol;Acc:HGNC:2681] | 8926 | -0.226 | -0.2030 | No |
| 36 | BAX | "BCL2 associated X, apoptosis regulator [Source:HGNC Symbol;Acc:HGNC:959]" | 9323 | -0.257 | -0.2168 | No |
| 37 | GRIN2C | glutamate ionotropic receptor NMDA type subunit 2C [Source:HGNC Symbol;Acc:HGNC:4587] | 9515 | -0.275 | -0.2134 | No |
| 38 | MAP3K5 | mitogen-activated protein kinase kinase kinase 5 [Source:HGNC Symbol;Acc:HGNC:6857] | 10923 | -0.421 | -0.2950 | Yes |
| 39 | CHP1 | calcineurin like EF-hand protein 1 [Source:HGNC Symbol;Acc:HGNC:17433] | 10940 | -0.423 | -0.2682 | Yes |
| 40 | TNFRSF1B | TNF receptor superfamily member 1B [Source:HGNC Symbol;Acc:HGNC:11917] | 10969 | -0.427 | -0.2421 | Yes |
| 41 | GRIN2D | glutamate ionotropic receptor NMDA type subunit 2D [Source:HGNC Symbol;Acc:HGNC:4588] | 11306 | -0.472 | -0.2369 | Yes |
| 42 | GRIN2B | glutamate ionotropic receptor NMDA type subunit 2B [Source:HGNC Symbol;Acc:HGNC:4586] | 11487 | -0.500 | -0.2178 | Yes |
| 43 | MAPK11 | mitogen-activated protein kinase 11 [Source:HGNC Symbol;Acc:HGNC:6873] | 11501 | -0.501 | -0.1856 | Yes |
| 44 | TOMM40 | translocase of outer mitochondrial membrane 40 [Source:HGNC Symbol;Acc:HGNC:18001] | 11977 | -0.589 | -0.1835 | Yes |
| 45 | MAP2K3 | mitogen-activated protein kinase kinase 3 [Source:HGNC Symbol;Acc:HGNC:6843] | 12004 | -0.593 | -0.1462 | Yes |
| 46 | GRIA1 | glutamate ionotropic receptor AMPA type subunit 1 [Source:HGNC Symbol;Acc:HGNC:4571] | 12149 | -0.631 | -0.1156 | Yes |
| 47 | GRIN1 | glutamate ionotropic receptor NMDA type subunit 1 [Source:HGNC Symbol;Acc:HGNC:4584] | 12603 | -0.799 | -0.0978 | Yes |
| 48 | PPP3R1 | "protein phosphatase 3 regulatory subunit B, alpha [Source:HGNC Symbol;Acc:HGNC:9317]" | 12653 | -0.829 | -0.0467 | Yes |
| 49 | APAF1 | apoptotic peptidase activating factor 1 [Source:HGNC Symbol;Acc:HGNC:576] | 12812 | -0.993 | 0.0068 | Yes |
Table: GSEA details [plain text format]

  

Fig 2: KEGG\_AMYOTROPHIC\_LATERAL\_SCLEROSIS\_ALS      
 Blue-Pink O' Gram in the Space of the Analyzed GeneSet

  

Fig 3: KEGG\_AMYOTROPHIC\_LATERAL\_SCLEROSIS\_ALS: Random ES distribution      
 Gene set null distribution of ES for **KEGG\_AMYOTROPHIC\_LATERAL\_SCLEROSIS\_ALS**
